# Supplementary material for: Effect of Artemisia absinthium and Malva sylvestris on Antioxidant Parameters and Abomasal Histopathology in Lambs Experimentally Infected with Haemonchus contortus
Source: Animals (Basel). 2021 Feb 9;11(2):462. doi: 10.3390/ani11020462 (PMC7916408; doi:10.3390/ani11020462)
Supplement: Supplementary file 1 [file animals-11-00462-s001.pdf]

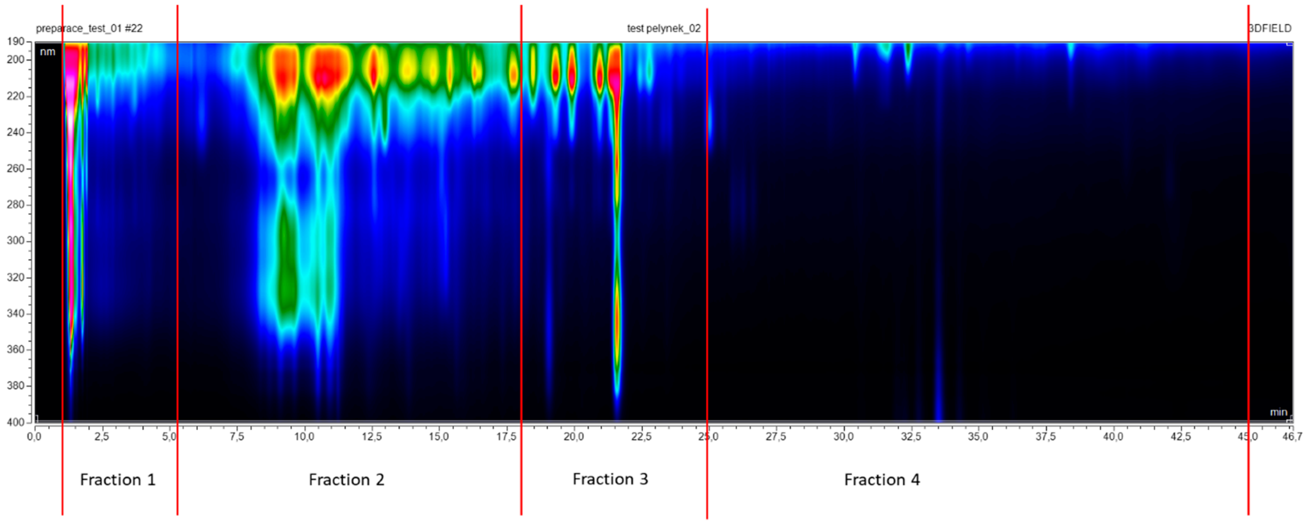

Figure S1. PDA chromatogram of *A. absinthium* methanolic extract with marked collected fractions (2D view, 190–400 nm).

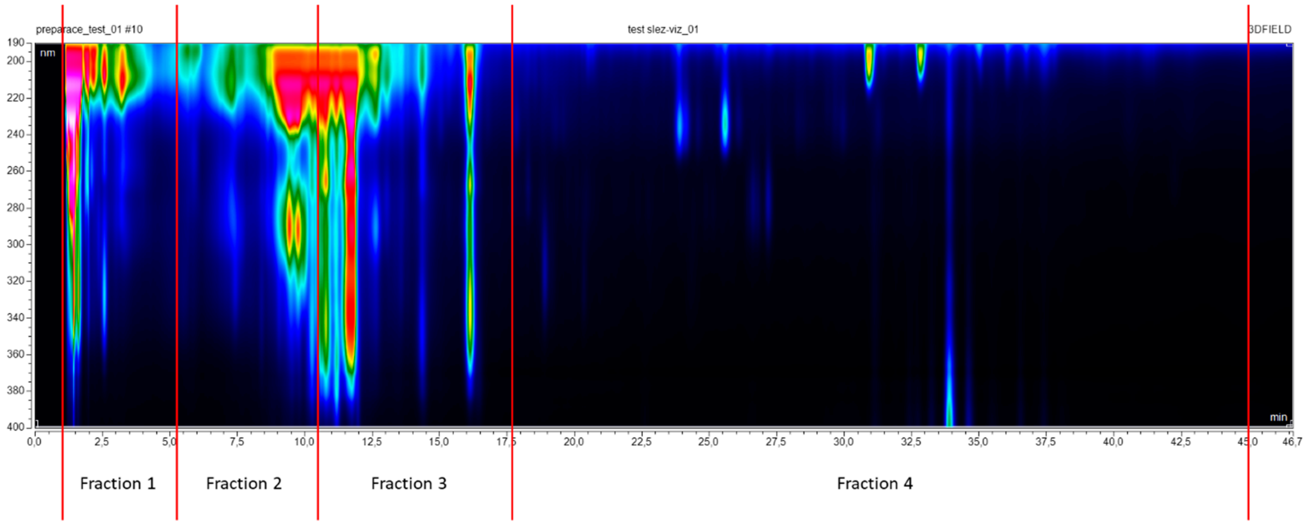

Figure S2. PDA chromatogram of *M. sylvestris* methanolic extract with marked collected fractions (2D view, 190–400 nm).

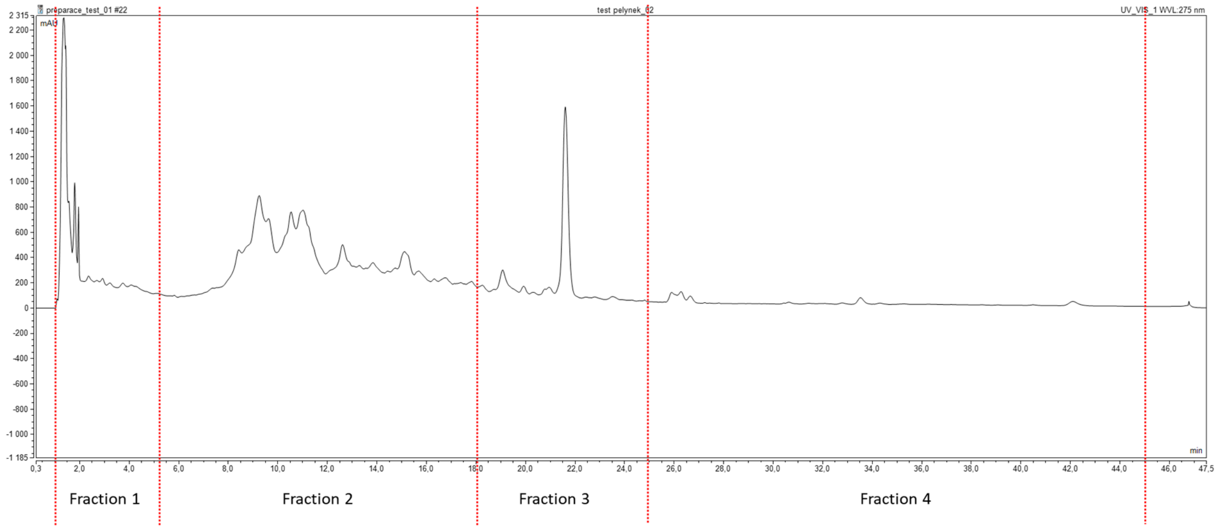

Figure S3. UV-Vis chromatogram of *A. absinthium* methanolic extract with marked collected fractions detected at 275 nm.

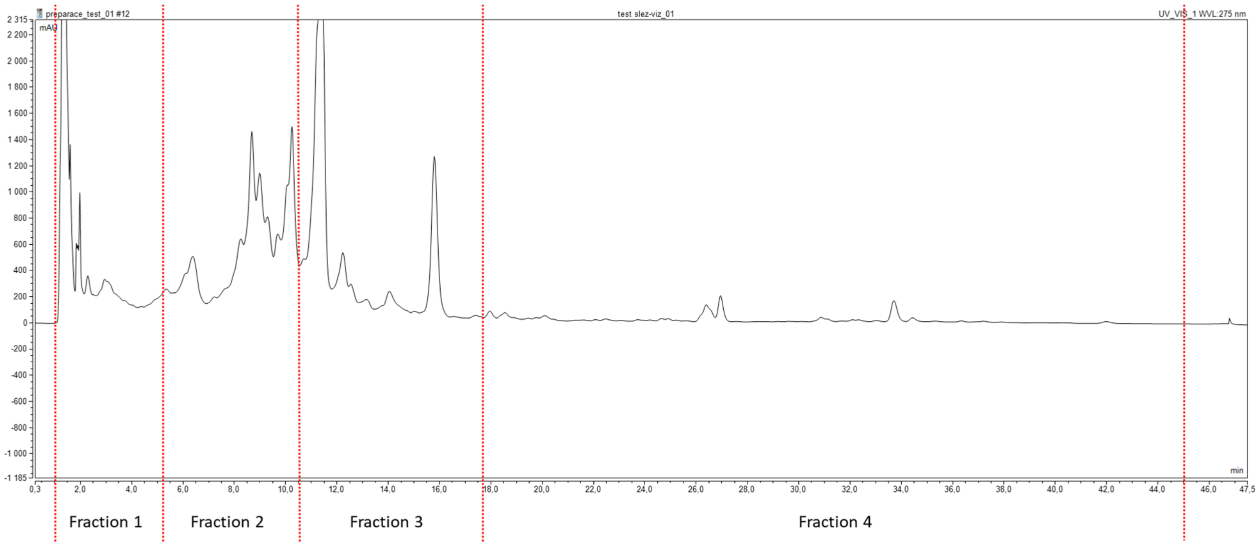

Figure S4. UV-Vis chromatogram of *M. sylvestris* methanolic extract with marked collected fractions detected at 275 nm.
